# Multiparametric MRI-based radiomics of whole-tumor and habitat regions for predicting HER2 status in young breast cancer: a two-center study

**DOI:** 10.3389/fonc.2026.1760589

**Published:** 2026-03-31

**Authors:** Xiaobin Jiang, Darong Zhu, Xumeng Luo, Siqing Fang, Nianshi Song, Qian Yu, Sicong Huang, Shiwei Wang

**Affiliations:** 1 Department of Radiology, The First Affiliated Hospital of Zhejiang Chinese Medical University (Zhejiang Provincial Hospital of Chinese Medicine), Hangzhou, Zhejiang, China; 2 The First School of Clinical Medicine, Zhejiang Chinese Medical University, Hangzhou, Zhejiang, China; 3 Department of Radiology, Hangzhou First People’s Hospital, Hangzhou, Zhejiang, China; 4 Clinical Science, Philips Healthcare, Beijing, China

**Keywords:** breast cancer, habitat, HER2, multiparametric magnetic resonance imaging, radiomics, young

## Abstract

**Objective:**

Habitat imaging can quantify intratumoral heterogeneity in young breast cancer patients, providing support for the prediction of HER2 expression levels. Therefore, this study aimed to compare the predictive ability of habitat models and conventional whole-tumor models for HER2 expression status in young breast cancer patients using multiparametric MRI.

**Methods:**

A retrospective cohort consisting of 375 young breast cancer patients (age < 40 years) who underwent preoperative MRI scanning at two medical centers was included in this study. Two binary classification tasks were designed: Task 1 (HER2 negative expression vs. HER2 positive expression) and Task 2 (HER2 zero expression vs. HER2 low expression). The training cohort (n=206) comprised patients from Center 1, with the external validation cohort (n=169) recruited from Center 2. Clinicopathological and MRI characteristics were collected. Radiomics features based on whole-tumor and habitat regions were extracted from DCE-MRI and DWI images, respectively. Clinical models, conventional whole-tumor models, habitat models, and combined models were constructed. Subsequently, model performance was evaluated by the AUC, sensitivity, and specificity.

**Results:**

In Task 1, the AUC of the clinical model, conventional whole-tumor model, habitat model, and combined model in the training cohort were 0.683, 0.731, 0.761, and 0.768 respectively. In Task 2, no clinicopathological features were determined as independent risk factors, thus no clinical model was developed. The AUC for the whole-tumor model, habitat model, and combined model in the training cohort were 0.673, 0.649, and 0.758 respectively.

**Conclusion:**

The habitat model exhibited better discriminatory effectiveness in identifying HER2 positive expression in young breast cancer patients, in comparison to the whole-tumor radiomics model. The integration of conventional whole-tumor radiomics features with habitat features and clinicopathological characteristics can enhance model performance.

## Introduction

Breast cancer, the most common malignant neoplasm among females, has shown an increasing incidence trend in younger age groups over recent years (1). In accordance with the latest International Consensus Guidelines on Breast Cancer in Young Women, young breast cancer patients are defined as patients under 40 years of age (2). Compared with older patients, young breast cancer patients frequently present poor prognostic clinicopathological features and increased tumor aggressiveness, exhibiting elevated expression of molecular biomarkers such as human epidermal growth factor receptor 2 (HER2) and the proliferation marker Ki-67 (3, 4). A Danish study demonstrated that patients aged 35–39 years presented a 40% elevated relative risk of mortality compared with those aged 45–49 years (5). This disparity is closely linked to the more aggressive biological behavior of breast cancer in young patients.

HER2 is a pivotal driver gene in breast cancer, exerting a significant influence on tumor invasiveness and patient prognosis (6). The DESTINY-Breast04 trial demonstrated for the first time that trastuzumab deruxtecan provides significant clinical benefits to patients with HER2-low metastatic breast cancer (7). This finding has brought new hope to the long-standing clinical dilemma of the absence of targeted therapies for HER2-low breast cancer, reshaped the overall landscape of anti-HER2 treatment for breast cancer, and also introduced new requirements for the re-evaluation and systematic optimization of clinical anti-HER2 treatment strategies. Currently, the determination of HER2 expression levels is predominantly achieved using combined immunohistochemistry (IHC) and fluorescence *in situ* hybridization (FISH) testing. However, due to the high heterogeneity of tumor tissue, IHC and FISH performed on partial tissue samples cannot fully reflect the HER2 expression levels within the tumor (8). Therefore, the use of a method that can globally characterize tumor heterogeneity to assess HER2 expression status carries important clinical significance for the precise diagnosis and individualized treatment of young breast cancer patients.

Magnetic resonance imaging (MRI) serves as an essential modality for the diagnosis of breast cancer and assessment of treatment efficacy. Among its applications, dynamic contrast-enhanced MRI (DCE-MRI) can assess tumor heterogeneity by characterizing the distribution of tumor microvessels through hemodynamic parameters (9). Traditional radiomics is a method that converts medical images into high-throughput quantitative features for quantitative analysis (10). Previous studies have confirmed that it holds substantial clinical value in predicting HER2 expression in breast cancer patients across all age groups (11–13). However, traditional radiomics has inherent limitations in effectively quantifying intratumoral heterogeneity. As an emerging branch of radiomics, habitats analysis clusters similar radiomics features in imaging data to partition tumors into subregions with distinct pathobiological characteristics, thereby revealing the spatial distribution of intratumoral heterogeneity (14). In recent years, numerous studies have demonstrated that, compared with conventional radiomics, habitat radiomics exhibits more significant advantages in predicting HER2 expression in breast cancer patients across all age groups (15–17).

To date, the application value of both traditional MRI radiomics and habitat radiomics in predicting HER2 expression status in breast cancer patients of all ages has been clinically validated. However, given that young breast cancer patients exhibit distinct clinical and biological characteristics, no studies have yet applied traditional radiomics or habitat radiomics models to this specific population, nor systematically compared the predictive performance of the two models for HER2 expression status in these patients. Therefore, we constructed clinical models, whole-tumor radiomics models, habitat models, and combined models respectively, based on dual-center data of young breast cancer patients. The primary objective of this study was to compare the discriminative efficacy between the conventional whole-tumor model and the habitat model in distinguishing HER2-positive from HER2-negative tumors in young breast cancer patients, and to further evaluate the model performance in differentiating HER2-low expression from HER2-zero expression among these patients.

## Materials and methods

### Patients

This retrospective multicenter study was approved by the ethics committees of the participating institutions (Approval No. 2021-KL-073–03 and 2025ZN485-1).

A total of 322 young breast cancer patients were enrolled from Center 1 between May 2013 and July 2024, and 226 young breast cancer patients from Center 2 between December 2013 and December 2024. The inclusion criteria were as follows: (1) pathologically confirmed young breast cancer patients (age < 40 years); (2) patients who had undergone standard postoperative IHC and FISH testing; (3) patients who had undergone standardized MRI scanning. The exclusion criteria comprised the following: (1) poor quality of MRI images; (2) patients with incomplete clinicopathological data; (3) patients who had undergone surgery or neoadjuvant therapy before MRI scanning.

**Figure 1:**
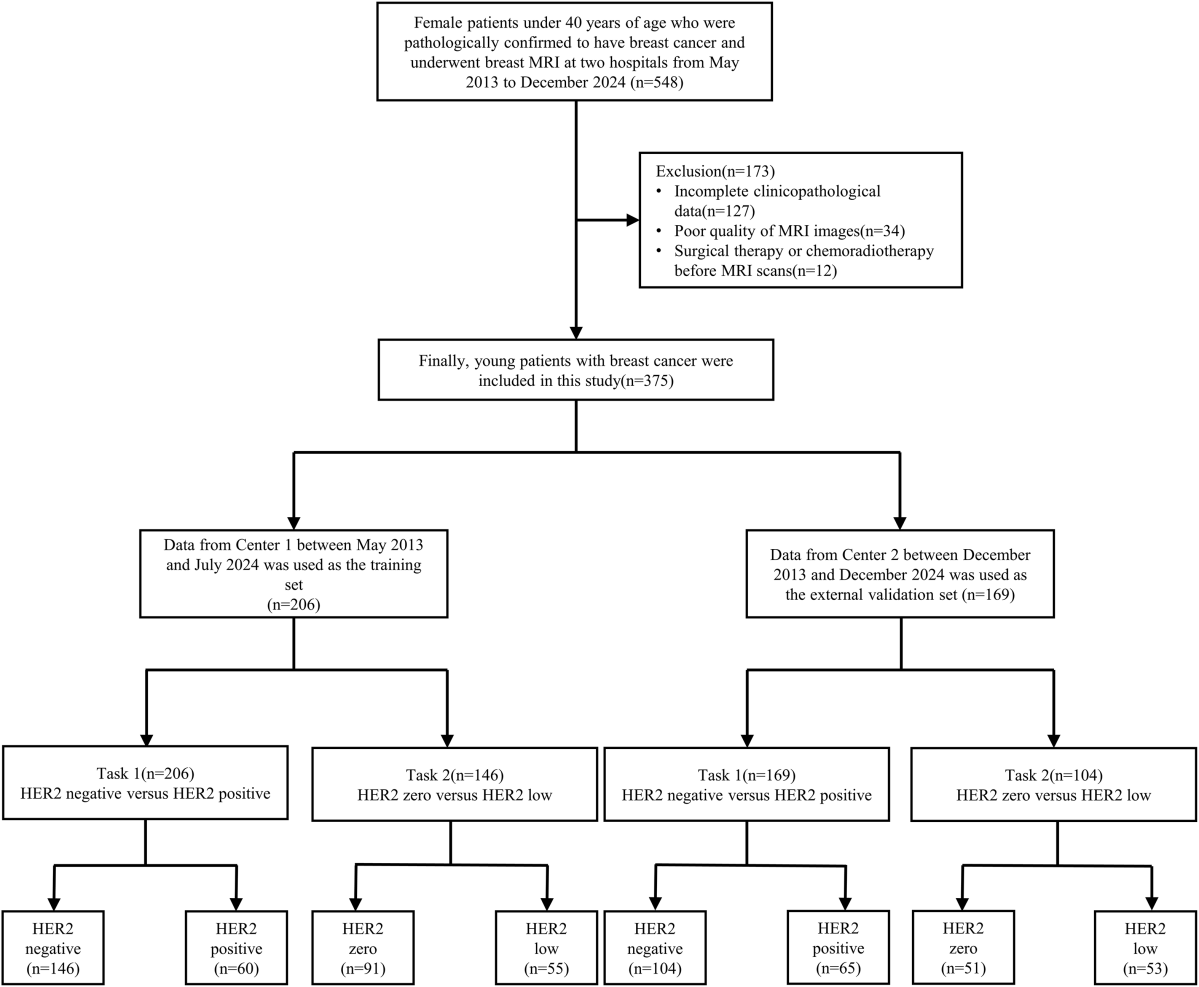
Flowchart depicting patient selection and grouping.

After retrospective analysis of clinicopathological data and MRI images, the following patients were excluded from the study: (1) Incomplete clinicopathological data (n=127); (2) Poor quality of MRI images (n=34); (3) Surgical therapy or chemoradiotherapy before MRI scans (n=12). Finally, 375 patients participated in this study. Based on pathological findings, two binary classification tasks were designed for young breast cancer patients with different HER2 expression statuses. Task 1 was HER2 negative (including HER2 zero and low expression) versus HER2 positive expression; Task 2 was HER2 zero expression versus HER2 low expression. A total of 206 patients recruited from Center 1 were assigned to the training cohort, while an external validation cohort was composed of 169 patients recruited from Center 2. **Figure 1** illustrates the flowchart of patient selection and grouping.

### MRI examination

Breast MRI data were acquired at two independent institutions. Center 1 performed imaging using a 3.0T MR scanner (Magnetom Verio, Siemens Healthcare, Erlangen, Germany) equipped with a 16-channel breast-specific coil, while Center 2 used a 1.5T MR scanner (Avanto, Siemens Healthcare, Erlangen, Germany) equipped with an 8-channel breast-specific coil. During the examination, patients adopted a prone position, with both breasts naturally suspended within the coil openings and the head oriented anteriorly. Diffusion-weighted imaging (DWI) images were acquired before the start of contrast-enhanced scanning. In DCE-MRI, a baseline non-contrast-enhanced image was obtained first, followed by five consecutive phases of scanning after contrast agent injection. Gadolinium diethylene-triamine pentoacetic acid was adopted as the contrast agent, administered at a dose of 0.1 mmol/kg. The agent was injected into the antecubital vein at an infusion rate of 2 ml/s, after which 14 ml of saline was delivered at the identical rate. Detailed parameters for MRI sequence acquisition are provided in the **Supplementary Material Table 1**.

### Clinicopathological and conventional MRI features

Clinicopathological data of each patient were collected, including age, tumor diameter, lymph node metastasis, histology type, estrogen receptor (ER) expression, progesterone receptor (PR) expression, and Ki-67 index. MRI data were collected and classified according to the 5th edition BI-RADS classification (18). Conventional MRI features included fibroglandular tissue, background parenchymal enhancement (BPE), enhancement pattern, internal enhancement, and time-intensity curve (TIC).

The HER2 expression status of young breast cancer patients was determined by standard IHC and FISH testing. The results were interpreted in accordance with the American Society of Clinical Oncology (ASCO) practice guidelines and the European Society for Medical Oncology (ESMO) expert consensus (8, 19). HER2 zero expression was defined as: IHC score of 0; HER2 low expression as: IHC score of 1+ or IHC 2+, without FISH amplification; HER2 positive expression as: IHC score of 2+, with FISH amplification or IHC 3 +. In line with the international breast cancer expert consensus on Ki-67 in breast cancer (20), this study used 14% as the threshold for evaluating Ki-67 expression status: Ki-67 ≥ 14% was classified as high expression, and Ki-67 < 14% was classified as low expression. ER and PR expression levels were assessed by immunohistochemistry, with ≥1% of tumor cells showing nuclear staining defined as positive and <1% as negative.

### Tumor segmentation and habitat generation

DWI and DCE-MRI images were selected and imported into 3D Slicer software (version 5.6.2, http://www.slicer.org/) for delineation. A radiology resident with three years of radiology experience, blinded to the pathological results, performed layer-by-layer semi-automated delineation of the tumor region of interest (ROI), covering the entire tumor area. The delineations were then independently reviewed by a radiology consultant with eight years of experience. In case of disagreement on ROI segmentation boundaries, a third radiologist with more than 20 years of experience was engaged to serve as an arbitrator and to revise the tumor ROI. The optimal number of intratumoral habitats was automatically determined using the Gaussian Mixture Model and the Bayesian Information Criterion. The tumor region was segmented into three subregions at the voxel level using the K-means clustering algorithm. To ensure inter-patient consistency, clustering was performed at the cohort level.

### Feature selection

Radiomics features were extracted from the habitat subregions and the whole-tumor area of the segmented lesion ROIs using a standardized pipeline. The extracted feature types included first-order statistical features, shape-based features, texture features, and higher-order features reflecting heterogeneity patterns. The extracted features complied with the Image Biomarker Standardization Initiative (21).

Subsequently, feature selection and dimensionality reduction were conducted for conventional radiomics features from the whole-tumor region and habitat features from the habitat subregion. Initially, Analysis of Variance (ANOVA) was employed to retain radiomics features with *P* < 0.05 only. Recursive Feature Elimination (RFE) was then applied to further eliminate feature redundancy. Finally, the least absolute shrinkage and selection operator (LASSO) was used to select the optimal subset of radiomics features. For clinicopathological and conventional MRI features, univariate logistic regression analysis was separately conducted for Task 1 and Task 2, retaining features with *P* < 0.05. Subsequently, multivariate logistic regression analysis was used to determine the independent risk factors for predicting HER2 expression status in young breast cancer patients.

### Model development and interpretation

**Figure 2:**
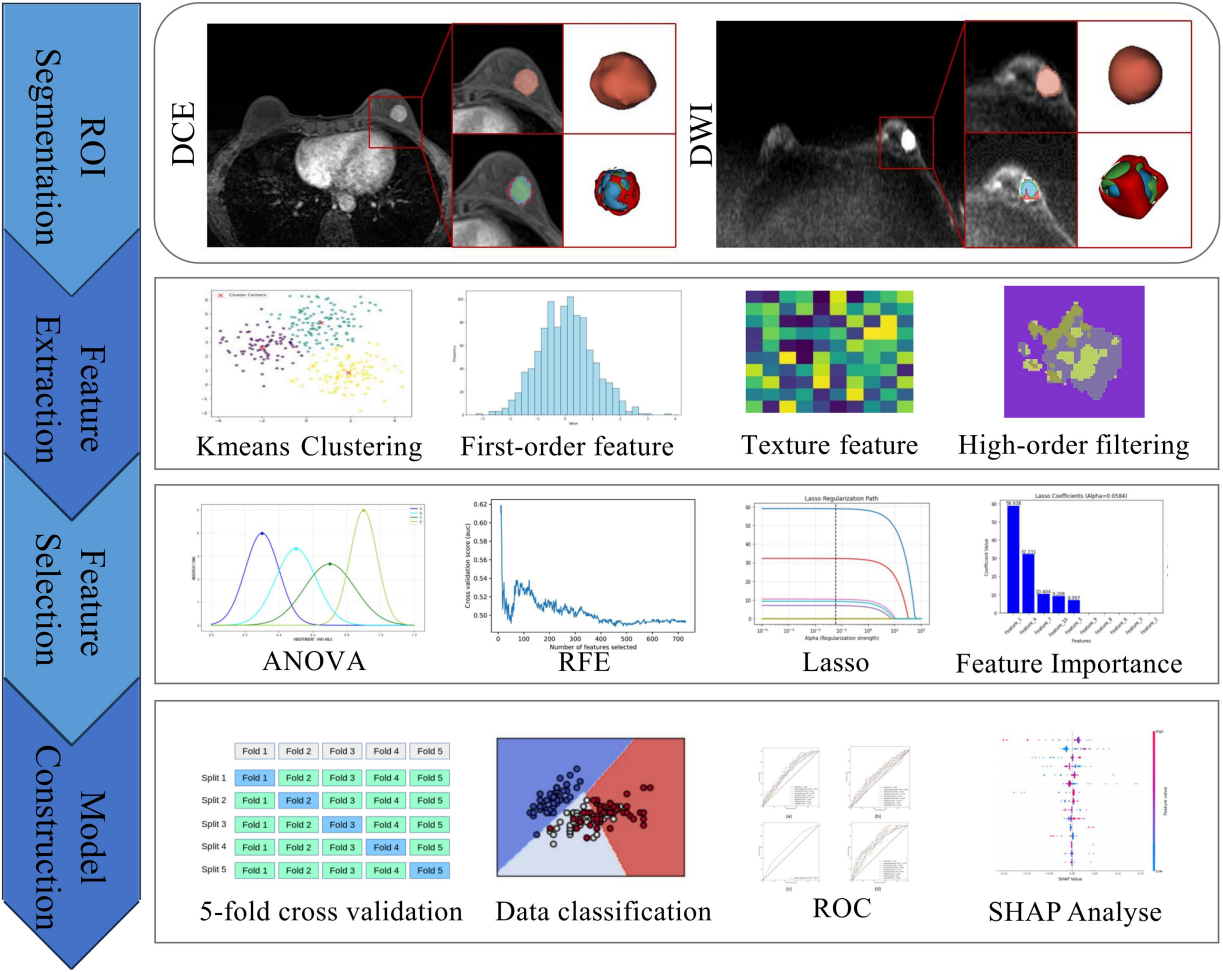
Workflow of medical image analysis. DCE, dynamic contrast-enhancement; DWI, diffusion weighted imaging; ANOVA, analysis of variance; RFE, recursive feature elimination; LASSO, least absolute shrinkage and selection operator; ROC, receiver operating characteristic; ROI, region of interest; SHAP, SHapley Additive exPlanations.

Data of young breast cancer patients from Center 1 were assigned to the training cohort, and internal validation was conducted through five-fold cross-validation of the training set from Center 1. Data from Center 2 were used exclusively for external independent validation. Clinicopathological and conventional MRI features identified as independent risk factors through univariate and multivariate logistic regression were selected for the development of clinical models. For classifier selection in the whole-tumor radiomics model and the habitat model, seven classification algorithms—Decision Tree, Random Forest, Extra Trees, Easy Ensemble, RUS Boost, XG Boost, and Light GBM—were used for model training. In addition, a combined model was developed by integrating selected features from the whole-tumor models, habitat models, and clinical models. **Figure 2** illustrates the workflow of medical image analysis.

After model construction, SHapley Additive exPlanations (SHAP) analysis was employed to visually interpret features of both the whole-tumor model and the habitat model, thereby improving the interpretability of the models.

### Statistical analysis

Continuous variables were analyzed using the Mann-Whitney U test or Student’s t-test. Categorical variables were analyzed using the chi-square test or Fisher’s exact test. Univariate and multivariate logistic regression analyses were used to identify independent risk factors within the clinical model. Model performance was evaluated using the area under the ROC curve (AUC) and corresponding 95% confidence interval (CI). Furthermore, two-sided *P*-values less than 0.05 were considered statistically significant. All statistical analyses were performed using Python software (version 3.9.13, http://www.python.org/).

## Results

### Clinicopathological and MRI characteristics

This study enrolled a total of 375 young breast cancer patients (mean age: 35.26 ± 3.74 years) from two centers. Among these, there were 142 patients with HER2 zero expression, 118 patients with HER2 low expression, and 115 patients with HER2 positive expression. For Task 1 and 2, statistical analysis was conducted on the clinicopathological and MRI characteristics of young breast cancer patients in both the training and external validation cohorts. For Task 1, compared with HER2-negative patients, HER2-positive patients had a higher proportion of Ki-67 high expression and PR negative expression in both the training and external validation cohorts. Tumor diameter, ER expression, enhancement pattern, and internal enhancement showed significant differences only in the training cohort (**Table 1**). For Task 2, young breast cancer patients with low HER2 expression demonstrated a higher proportion of PR-positive expression in both the training and external validation cohorts compared to those with zero HER2 expression. ER expression was statistically significant only in the external validation cohort (**Table 2**).

**Table 1:** Clinicopathologic and MRI characteristics of study patients in task 1 (HER2-negative vs HER2-positive).

Characteristics	Training			Test		
	HER2-negative (n=146)	HER2-positive (n=60)	*P* value	HER2-negative (n=104)	HER2-positive (n=65)	*P* value
Age​	35.49 ± 3.73	34.75 ± 4.34	0.2943	35.46 ± 3.52	34.91 ± 3.53	0.2770
Tumor Diameter (cm)	2.40 ± 1.38	2.96 ± 1.48	0.0034	2.87 ± 1.68	3.00 ± 1.80	0.6253
Fibroglandular Tissue			0.7486			0.6028
Fatty	1 (0.68%)	1 (1.67%)		0 (0.00%)	1 (1.54%)	
Scattered Fibroglandular	1 (0.68%)	1 (1.67%)		5 (4.81%)	4 (6.15%)	
Heterogeneously Dense	135 (92.47%)	53 (88.33%)		92 (88.46%)	55 (84.62%)	
Extremely Dense	9 (6.16%)	5 (8.33%)		7 (6.73%)	5 (7.69%)	
Lymph Node Metastasis			0.3708			0.1242
Negative	96 (65.75%)	44 (73.33%)		65 (62.50%)	32 (49.23%)	
Positive	50 (34.25%)	16 (26.67%)		39 (37.50%)	33 (50.77%)	
Histology Type​			0.1771			0.0691
Invasive Carcinoma	125 (85.62%)	46 (76.67%)		91 (87.50%)	63 (96.92%)	
Others	21 (14.38%)	14 (23.33%)		13 (12.50%)	2 (3.08%)	
ER Expression			0.0182			0.2029
Negative, <1%	29 (19.86%)	22 (36.67%)		26 (25.00%)	23 (35.38%)	
Positive, ≥1%	117 (80.14%)	38 (63.33%)		78 (75.00%)	42 (64.62%)	
PR Expression			0.0453			0.0488
Negative, <1%	28 (19.18%)	20 (33.33%)		24 (23.08%)	25 (38.46%)	
Positive, ≥1%	118 (80.82%)	40 (66.67%)		80 (76.92%)	40 (61.54%)	
Ki-67 Index			0.0017			0.0149
Low, <14%	47 (32.19%)	6 (10.00%)		35 (33.65%)	10 (15.38%)	
High, ≥14%	99 (67.81%)	54 (90.00%)		69 (66.35%)	55 (84.62%)	
BPE			0.1662			0.4081
Minimal or Mild	58 (39.73%)	17 (28.33%)		64 (61.54%)	35 (53.85%)	
Moderate or Severe	88 (60.27%)	43 (71.67%)		40 (38.46%)	30 (46.15%)	
Enhancement Pattern			0.0132			0.2867
Mass	116 (79.45%)	37 (61.67%)		78 (75.00%)	43 (66.15%)	
Non-Mass	30 (20.55%)	23 (38.33%)		26 (25.00%)	22 (33.85%)	
Internal Enhancement			0.0226			0.8137
Homogeneous	52 (35.62%)	11 (18.33%)		18 (17.31%)	13 (20.00%)	
Heterogeneous	94 (64.38%)	49 (81.67%)		86 (82.69%)	52 (80.00%)	
TIC			0.5489			0.6873
I	15 (10.27%)	7 (11.67%)		6 (5.77%)	2 (3.08%)	
II	21 (14.38%)	12 (20.00%)		55 (52.88%)	37 (56.92%)	
III	110 (75.34%)	41 (68.33%)		43 (41.35%)	26 (40.00%)	

ER, estrogen receptor; PR, progesterone receptor; HER-2, human epidermal growth factor receptor-2; BPE, background parenchymal enhancement; TIC, time intensity curve.

**Table 2:** Clinicopathologic and MRI characteristics of study patients in task 2 (HER2-zero vs HER2-low).

Characteristics	Training			Test		
	HER2-zero (n=91)	HER2- low (n=55)	*P* value	HER2-zero (n=51)	HER2- low (n=53)	*P* value
​Age​	35.42 ± 3.76	35.62 ± 3.70	0.9072	35.12 ± 3.61	35.79 ± 3.43	0.3076
Tumor Diameter (cm)	2.41 ± 1.38	2.39 ± 1.39	1.0000	2.79 ± 1.48	2.95 ± 1.87	0.9766
Fibroglandular Tissue			0.3088			0.0694
Fatty	0 (0.00%)	1 (1.82%)		0 (0.00%)	0 (0.00%)	
Scattered Fibroglandular	0 (0.00%)	1 (1.82%)		4 (7.84%)	1 (1.89%)	
Heterogeneously Dense	86 (94.51%)	49 (89.09%)		46 (90.20%)	46 (86.79%)	
Extremely Dense	5 (5.49%)	4 (4 (7.27%) 7.14%)		1 (1.96%)	6 (11.32%)	
Lymph Node Metastasis			0.4006			0.8792
Negative	57 (62.64%)	39 (70.91%)		31 (60.78%)	34 (64.15%)	
Positive	34 (37.36%)	16 (29.09%)		20 (39.22%)	19 (35.85%)	
Histology Type​			0.4393			0.2075
Invasive Carcinoma	80 (87.91%)	45 (81.82%)		42 (82.35%)	49 (92.45%)	
Others	11 (12.09%)	10 (18.18%)		9 (17.65%)	4 (7.55%)	
ER Expression			0.0582			0.0004
Negative, <1%	23 (25.27%)	6 (10.91%)		21 (41.18%)	5 (9.43%)	
Positive, ≥1%	68 (74.73%)	49 (89.09%)		30 (58.82%)	48 (90.57%)	
PR Expression			0.0285			0.0076
Negative, <1%	23 (25.27%)	5 (9.09%)		18 (35.29%)	6 (11.32%)	
Positive, ≥1%	68 (74.73%)	50 (90.91%)		33 (64.71%)	47 (88.68%)	
Ki-67 Index			0.5118			0.2689
Low, <14%	27 (29.67%)	20 (36.36%)		14 (27.45%)	21 (39.62%)	
High, ≥14%	64 (70.33%)	35 (63.64%)		37 (72.55%)	32 (60.38%)	
BPE			0.6377			0.7213
Minimal or Mild	38 (41.76%)	20 (36.36%)		30 (58.82%)	34 (64.15%)	
Moderate or Severe	53 (58.24%)	35 (63.64%)		21 (41.18%)	19 (35.85%)	
Enhancement Pattern			0.9331			0.9098
Mass	73 (80.22%)	43 (78.18%)		39 (76.47%)	39 (73.58%)	
Non-Mass	18 (19.78%)	12 (21.82%)		12 (23.53%)	14 (26.42%)	
Internal Enhancement			0.1447			0.2276
Homogeneous	37 (40.66%)	15 (27.27%)		6 (11.76%)	12 (22.64%)	
Heterogeneous	54 (59.34%)	40 (72.73%)		45 (88.24%)	41 (77.36%)	
TIC			0.9352			0.4213
I	10 (10.99%)	5 (9.09%)		4 (7.84%)	2 (3.77%)	
II	13 (14.29%)	8 (14.55%)		24 (47.06%)	31 (58.49%)	
III	68 (74.73%)	42 (76.36%)		23 (45.10%)	20 (37.74%)	

ER, estrogen receptor; PR, progesterone receptor; HER-2, human epidermal growth factor receptor-2; BPE, background parenchymal enhancement; TIC, time intensity curve.

### Feature selection

The tumor region was divided into three subregions by clustering tumor habitats at the voxel level using the K-means clustering algorithm. Subsequently, feature selection and dimensionality reduction were performed to obtain the optimal feature subset in each model. For Task 1, the 15 whole-tumor features and 13 significantly correlated habitat features were selected. In Task 2, 11 whole-tumor features were selected to develop the whole-tumor model, and 15 habitat features were selected to develop the habitat model. The detailed selected features are provided in **Supplementary Material Table 2**.

In Task 1, univariate logistic regression analysis of clinicopathological and conventional MRI features in the training cohort revealed significant correlations between several variables and HER2 expression in young breast cancer patients: tumor diameter (odds ratio [OR], 6.56 [95% CI: 1.49, 28.81]; *P* = 0.01), ER expression (OR, 0.43 [95% CI: 0.22, 0.83]; *P* = 0.01), PR expression (OR, 0.47 [95% CI: 0.24, 0.93]; *P* = 0.03), Ki-67 index (OR, 4.27 [95% CI: 1.72, 10.64]; *P*<0.01), enhancement pattern (OR, 2.40 [95% CI: 1.25, 4.64]; *P* = 0.01), and internal enhancement (OR, 2.46 [95% CI: 1.18, 5.15]; *P* = 0.02). Subsequently, multivariate logistic regression analysis of these variables identified Ki-67 index (OR, 4.10 [95% CI: 1.55, 10.86]; *P*<0.01) and enhancement pattern (OR, 2.27 [95% CI: 1.04, 4.95]; *P* = 0.04) as independent risk factors for predicting HER2 status in young breast cancer patients (**Table 3**). In Task 2, the same univariate and multivariate logistic regression analyses failed to identify any independent risk factors significantly associated with HER2-zero or low-expression status in young breast cancer patients (**Table 4**).

**Table 3:** Univariable and multivariable analyses of clinicopathological and MRI characteristics in task 1.

Variables	Univariable analysis		Multivariable analysis	
	OR (95%CI)	*P* value	OR (95%CI)	*P* value
Ki67 Index	4.27 (1.72, 10.64)	<0.01	4.10 (1.55, 10.86)	<0.01
Enhancement Pattern	2.40 (1.25, 4.64)	0.01	2.27 (1.04, 4.95)	0.04
ER Expression	0.43 (0.22, 0.83)	0.01	0.60 (0.16, 2.20)	0.44
Tumor Diameter	6.56 (1.49, 28.81)	0.01	1.46 (0.25, 8.43)	0.67
Internal Enhancement	2.46 (1.18, 5.15)	0.02	1.80 (0.80, 4.01)	0.15
PR Expression	0.47 (0.24, 0.93)	0.03	1.20 (0.32, 4.58)	0.79
Histology Type	1.81 (0.85, 3.86)	0.12		
BPE	1.67 (0.87, 3.20)	0.12		
Age	0.47 (0.14, 1.56)	0.22		
Lymph Node Metastasis	0.70 (0.36, 1.36)	0.29		
TIC	0.83 (0.54, 1.29)	0.41		
Fibroglandular Tissue	0.93 (0.39, 2.26)	0.88		

ER, estrogen receptor; PR, progesterone receptor; HER-2, human epidermal growth factor receptor-2; BPE, background parenchymal enhancement; TIC, time intensity curve.

**Table 4:** Univariable and multivariable analyses of clinicopathological and MRI characteristics in task 2.

Variables	Univariable analysis		Multivariable analysis	
	OR (95%CI)	*P* value	OR (95%CI)	*P* value
PR Expression	3.38 (1.20, 9.51)	0.02	3.06 (0.52, 18.02)	0.22
ER Expression	2.76 (1.05, 7.29)	0.04	1.13 (0.21, 6.12)	0.89
Internal Enhancement	1.83 (0.88, 3.78)	0.10		
Lymph Node Metastasis	0.69 (0.33, 1.41)	0.31		
Histology Type	1.62 (0.64, 4.10)	0.31		
Ki67 Index	0.74 (0.36, 1.50)	0.40		
Fibroglandular Tissue	0.67 (0.22, 2.07)	0.49		
BPE	1.25 (0.63, 2.50)	0.52		
Age	1.25 (0.32, 4.87)	0.75		
TIC	1.09 (0.65, 1.82)	0.75		
Enhancement Pattern	1.13 (0.50, 2.57)	0.77		
Tumor Diameter	0.95 (0.17, 5.40)	0.96		

ER, estrogen receptor; PR, progesterone receptor; HER-2, human epidermal growth factor receptor-2; BPE, background parenchymal enhancement; TIC, time intensity curve.

### Model performance and interpretation

#### Task 1: differentiating HER2-negative versus HER2-positive cancer

**Figure 3:**
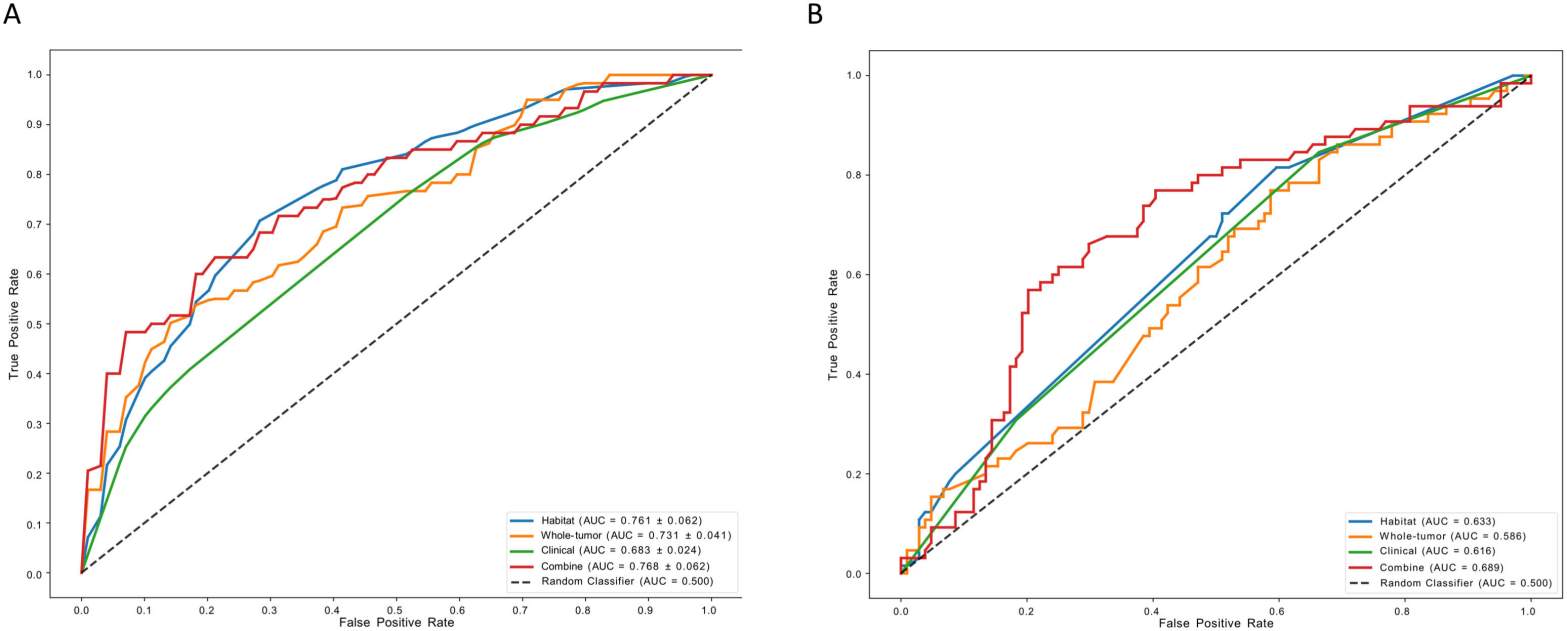
The performance of models in task 1. **(A)** represent AUCs of each model in the training cohorts; **(B)** represent AUCs of each model in the external validation cohorts. AUC, area under the receiver operating characteristic curve.

Using the two independent risk factors—enhancement pattern and Ki-67 index—as the features of the clinical model, the clinical model achieved an AUC of 0.683 (95% CI: 0.653, 0.712) and 0.616 (95% CI: 0.541, 0.698) in the training cohort and external validation cohort, respectively. The Extra Trees classification algorithm demonstrated superior performance compared to other classifiers in constructing the whole-tumor model and habitat model (**Supplementary Material Figure 1**). In the training and external validation cohorts, the AUC for the whole-tumor model was 0.731 (95% CI: 0.680, 0.782) and 0.586 (95% CI: 0.496, 0.668), respectively. The habitat model achieved an AUC of 0.761 (95% CI: 0.685, 0.839) and 0.633 (95% CI: 0.552, 0.713) in the training and external validation cohorts, respectively. In addition, by combining clinical features, whole-tumor features, and habitat features, the combined model exhibited the best discriminative performance among all models for HER2 status prediction, with AUCs of 0.768 (95% CI: 0.692, 0.845) and 0.689 (95% CI: 0.607, 0.774) in the training and external validation cohorts, respectively (**Figure 3**, and **Table 5**).

**Table 5:** Model performance in HER2 status prediction tasks.

Model	Training Set (5-fold CV)			External Validation Set		
	AUC (95% CI)	Sensitivity	Specificity	AUC (95% CI)	Sensitivity	Specificity
Task 1: HER2-Negative vs HER2-Positive						
Habitat Model	0.7615 (0.6845-0.8385)	0.6667	0.7117	0.6330 (0.5522-0.7131)	0.7231	0.4904
Whole-Tumor Model	0.7314 (0.6805-0.7823)	0.6167	0.6841	0.5864 (0.4959-0.6680)	0.3846	0.6923
Clinical Model	0.6825 (0.6529-0.7122)	0.1667	0.9246	0.6161 (0.5408-0.6978)	0.3077	0.8173
Combined Model	0.7685 (0.6918-0.8451)	0.6333	0.7184	0.6894 (0.6077-0.7742)	0.5692	0.7981
Task 2: HER2-Zero vs HER2-Low expression						
Habitat Model	0.6485 (0.4870-0.8101)	0.4364	0.7474	0.6160 (0.5046-0.7293)	0.5094	0.6863
Whole-Tumor Model	0.6727 (0.6370-0.7083)	0.4727	0.7374	0.6093 (0.4970-0.7233)	0.6415	0.5882
Clinical Model	–	–	–	–	–	–
Combined Model	0.7579 (0.6119-0.9040)	0.5273	0.8339	0.6323 (0.5181-0.7345)	0.3962	0.7647

**Figure 4:**
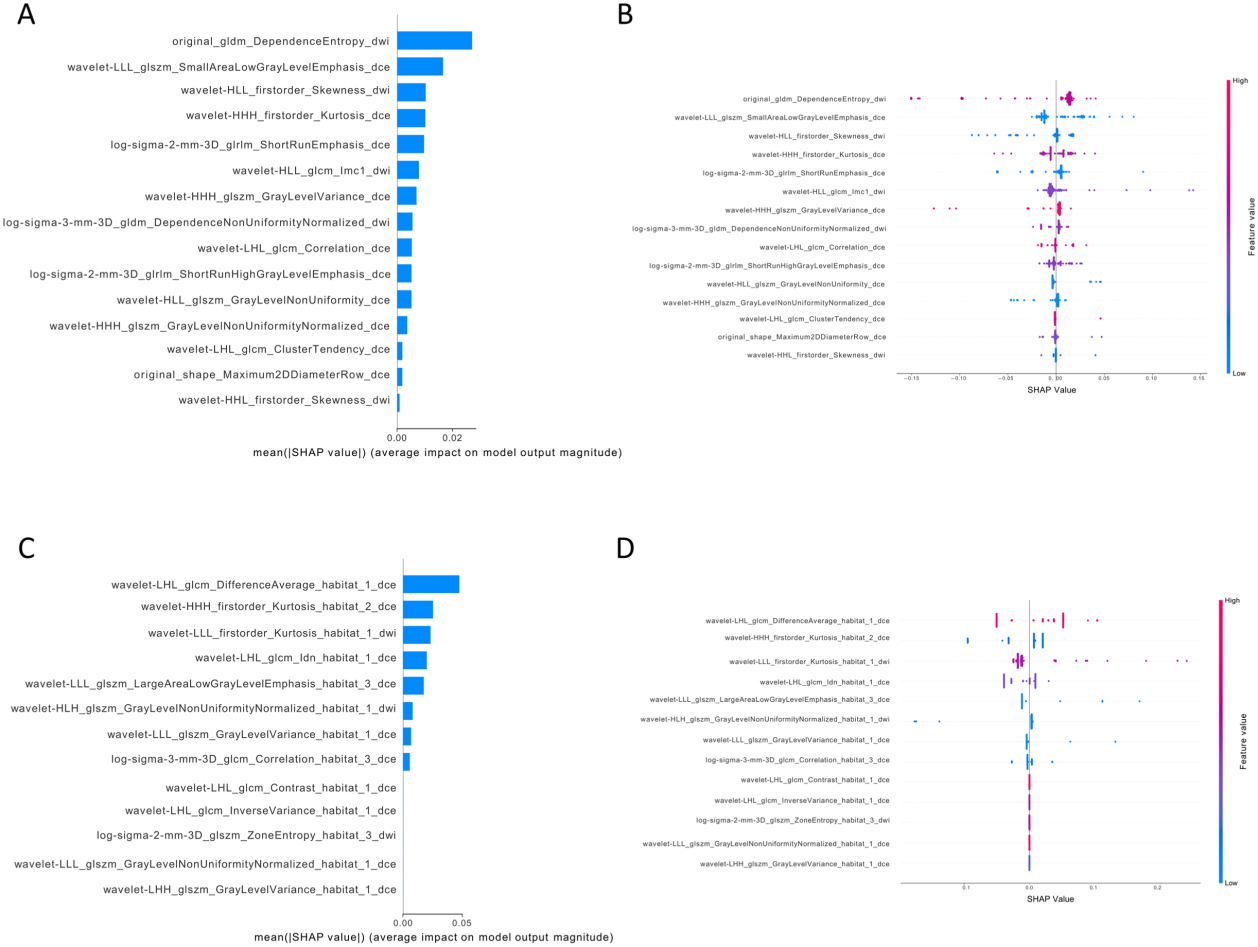
The interpretation of whole-tumor features and habitat features in task 1. **(A)** the bar plots illustrated the weight of the fifteen important features of the whole-tumor model; **(B)** the bar plots illustrated the weight of the thirteen important features of the habitat model. The horizontal axis represents the average shapley values, while the vertical axis represents the radiomics features. **(C)** the beeswarm plots revealed the relative importance of whole-tumor features and illustrate their actual relationships with the prediction outcomes. **(D)** the beeswarm plots revealed the relative importance of habitat features and illustrate their actual relationships with the prediction outcomes. Each individual is represented by a single dot on each feature flow. The horizontal position of the dot is determined by the SHAP value of that feature, and dots accumulated along each feature row to show density. SHAP, shapley additive explanations analysis.

The SHAP analysis revealed the contribution of whole-tumor radiomics features and habitat features to distinguishing HER2-negative from HER2-positive expression status in young breast cancer patients. The most significant influence among all features was exhibited by the *DifferenceAverage*, which is indicative of tumor heterogeneity. The majority of radiomics features derived from habitat regions have a positive effect on the model’s prediction results, while the positive impact of conventional radiomics features from the whole tumor on the model’s prediction results is limited (**Figure 4**).

#### Task 2: Differentiating between HER2-zero and HER2-low cancer

**Figure 5:**
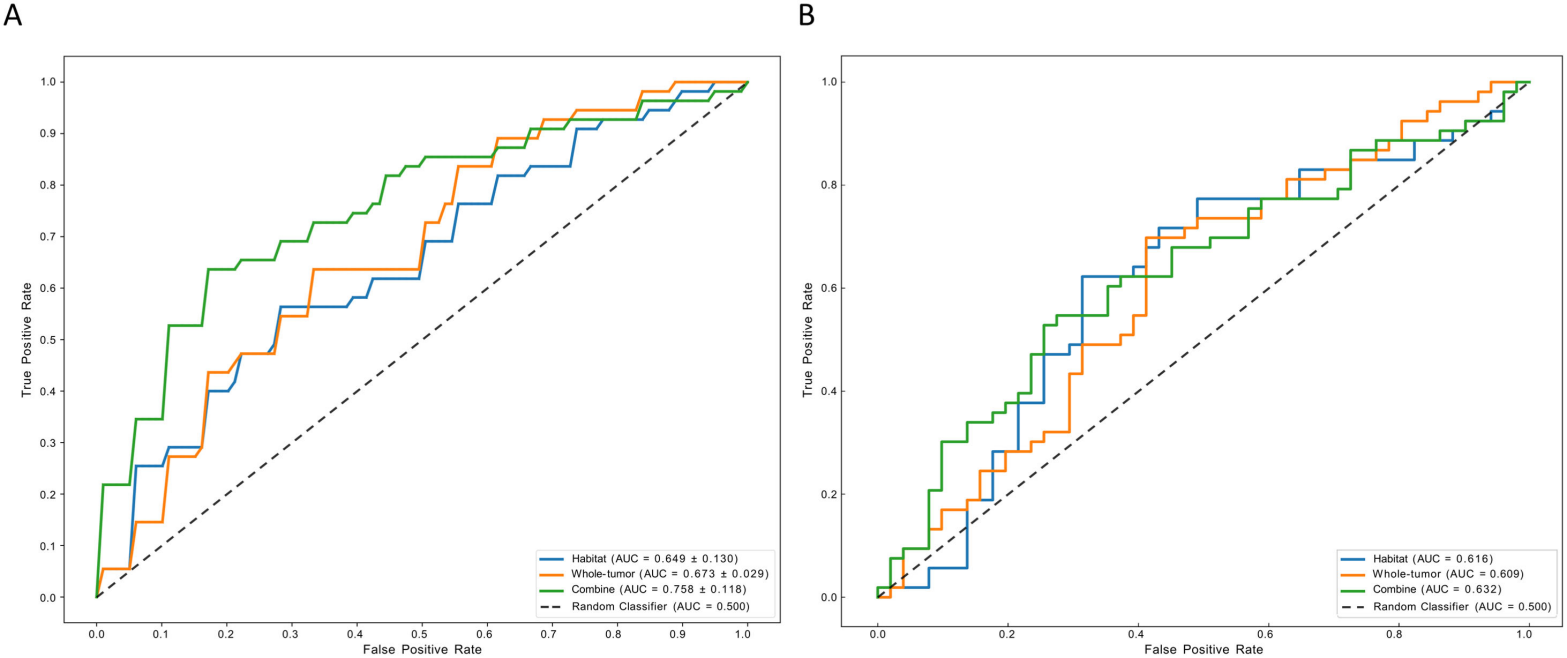
**(a)**: The performance of models in task 2. **(A)** represent AUCs of each model in the training cohorts; **(B)** represent AUCs of each model in the external validation cohorts. AUC, area under the receiver operating characteristic curve.

Univariate and multivariate logistic regression analyses of clinicopathological characteristics failed to identify independent risk factors, and therefore, the development of the clinical model was abandoned. Random Forest classification algorithm was selected as the classifier for the whole-tumor radiomics model, while the XG Boost classification algorithm outperformed other classifiers in the habitat model (**Supplementary Material Figure 1**). In the training and external validation cohorts, the AUC for the whole-tumor radiomics model was 0.673 (95% CI: 0.637, 0.708) and 0.609 (95% CI: 0.497, 0.723), respectively. The habitat model achieved an AUC of 0.649 (95% CI: 0.487, 0.810) and 0.616 (95% CI: 0.504, 0.729) in the training and external validation cohorts, respectively. Furthermore, the combined model demonstrated superior performance compared to both the whole-tumor radiomics model and the habitat model in both training and validation cohorts, achieving an AUC of 0.758 (95% CI: 0.611, 0.904) and 0.632 (95% CI: 0.518, 0.734), respectively (**Figure 5**, and **Table 5**).

**Figure 6:**
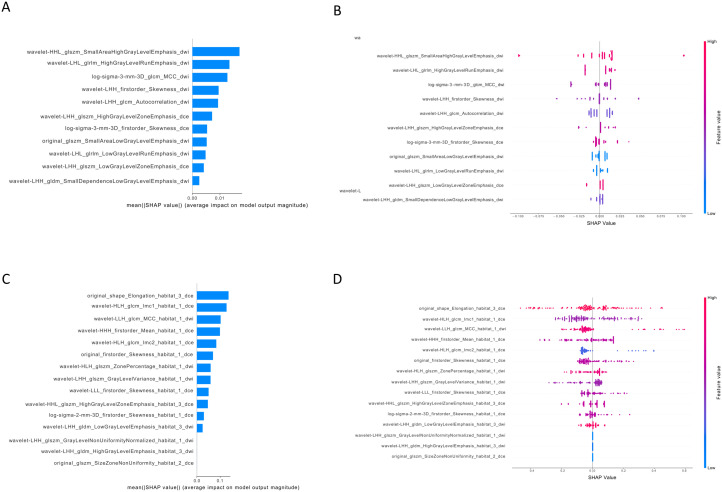
The interpretation of whole-tumor features and habitat features in task 2. **(A)** the bar plots illustrated the weight of the eleven important features of the whole-tumor model; **(B)** the bar plots illustrated the weight of the fifteen important features of the habitat model. The horizontal axis represents the average shapley values, while the vertical axis represents the radiomics features. **(C)** the beeswarm plots revealed the relative importance of whole tumor features and illustrate their actual relationships with the prediction outcomes. **(D)** the beeswarm plots revealed the relative importance of habitat features and illustrate their actual relationships with the prediction outcomes. Each individual is represented by a single dot on each feature flow. The horizontal position of the dot is determined by the SHAP value of that feature, and dots accumulated along each feature row to show density. SHAP, shapley additive explanations analysis.

The SHAP analysis (**Figures 6a–d**) indicated that the feature importance of characteristics derived from the whole tumor or habitat regions was comparable. The positive impact of features constructed for the whole-tumor radiomics model on prediction results was limited. However, radiomics features derived from the habitat region showed greater variability in their impact on model predictions, thereby reducing the output performance of the model.

## Discussion

This study aims to explore the clinical value of a multiparametric MRI radiomics model that relies on habitat imaging in predicting the status of HER2 expression among young breast cancer patients. We hypothesized that the heterogeneity of HER2 expression in breast cancer could lead to differences in both lesion signal intensity and internal enhancement characteristics across different tumor regions on MRI images. Habitat imaging technology can identify similar imaging features in complex and variable information for clustering, thereby quantifying intratumoral heterogeneity to better assist in predicting HER2 expression status (22). The results confirmed that the combined model incorporating habitat features (AUC, Task 1: 0.768, Task 2: 0.758) exhibited superior predictive performance in comparison to the whole-tumor radiomics model (AUC, Task 1: 0.731, Task 2: 0.673) in both classification tasks of HER2 expression status. This model achieved the highest AUC, indicating the greatest predictive efficacy among all models. This indicates that the habitat model can capture the spatial heterogeneity of intratumoral HER2 expression that cannot be reflected by traditional local tissue-based IHC/FISH testing. By globally characterizing tumor heterogeneity, the habitat model helps clinicians evaluate the overall biological behavior of tumors, predict the response to anti-HER2 therapy, and adjust treatment regimens in a timely manner for patients with heterogeneous HER2 expression.

It should also be noted that, compared with previous studies focusing on predicting HER2 expression status in breast cancer patients across all age groups, both the traditional radiomics model and the habitat model established in this study exhibited relatively lower performance in differentiating HER2 expression status among young breast cancer patients. We speculate that this observed discrepancy may be closely associated with the heterogeneity of tumor biological characteristics among breast cancer patients of different age groups.

For breast cancer patients covering all age groups, the wide age span gives rise to inherent heterogeneity in tumor biological behavior, clinicopathological features, and intratumoral microenvironment (23). Such inherent heterogeneity enables radiomic features to more clearly capture the differences in HER2 expression status. In contrast, tumors in young breast cancer patients present unique pathophysiological characteristics, which are specifically reflected in more aggressive biological behavior, more extensive tumor necrosis, and higher intratumoral heterogeneity (24, 25). These complex pathological structures and biological features significantly increase noise interference and instability in imaging features, making it challenging for radiomic features to accurately and stably reflect the HER2 expression status in young breast cancer patients and ultimately compromising the classification performance of the constructed models.

Previous studies have evaluated the performance of MRI radiomics models in distinguishing HER2 expression status in breast cancer (13, 26–29). Chen et al. (15) developed a habitat-based MRI radiomics model to identify HER2 statuses in breast cancer patients of all ages by quantifying intratumoral heterogeneity. This model outperformed conventional radiomics models in distinguishing HER2-positive from HER2-negative tumors across all cohorts. The separately constructed habitat model also achieved favorable performance in differentiating HER2-low from HER2-zero tumors. Li et al. (16) constructed deep learning and habitat models based on DCE MRI sequences, verifying that habitat features can noninvasively predict preoperative HER2 expression status in breast cancer patients through two prediction tasks. However, Chen et al. primarily focused on evaluating the performance of habitat-based MRI radiomics for predicting HER2 expression status in breast cancer. Notably, they did not compare the predictive efficacy between traditional radiomics models and habitat-based models for Task 2, which aimed to differentiate HER2-zero from HER2-low expression. In contrast, Li et al. integrated habitat analysis with deep learning approaches to predict HER2 status by extracting deep learning features from habitat subregions. Nevertheless, the inherent “black-box” characteristic of deep learning features resulted in relatively limited biological interpretability. Furthermore, these studies enrolled breast cancer patients of all age groups without specifically focusing on young breast cancer patients. Currently, there are few classification studies on HER2 expression status for this population. The term “young” has attracted increased attention in the clinical management of breast cancer patients. This is not only due to the biological challenges faced by this cohort, including poor differentiation, increased susceptibility to early metastasis, and a higher proportion of HER2-positive tumors, but also due to the need to consider the impact of treatment-related side effects on long-term survival (30, 31). Therefore, accurate HER2 assessment is crucial for implementing stratified management and personalized treatment strategies for young breast cancer patients.

In Task 1, the habitat model (AUC: 0.761, sensitivity: 66.7%, specificity: 71.2%) showed better performance than the whole-tumor radiomics model (AUC: 0.731, sensitivity: 61.7%, specificity: 68.4%) in distinguishing HER2-negative from HER2-positive expression in young breast cancer patients. This may be because habitat analysis focuses more on the similarities in signal intensity among different subregions within the tumor, thereby helping better reflect tumor microenvironment heterogeneity (32). HER2 amplification drives the abnormal proliferation and invasion of breast cancer cells, and this oncogenic signaling pathway further remodels the tumor microenvironment in a spatial heterogeneity manner (33). On the one hand, HER2-positive tumor subregions exhibit enhanced angiogenesis with higher microvessel density and increased vascular permeability, which is manifested as heterogeneous enhancement on DCE-MRI and corresponding changes in diffusion characteristics on DWI. These pathobiological changes are precisely captured by habitat subregions with distinct signal intensity and texture features (34). On the other hand, the high aggressiveness of HER2-positive tumors leads to uneven intratumoral necrosis and stromal reaction, forming tumor subregions with different cellular density and extracellular matrix composition (35). Habitat analysis can distinguish these subregions from the viable tumor parenchyma, and the radiomic features extracted from these heterogeneous subregions essentially represent the spatial variation of tumor biological behavior regulated by HER2. Collectively, the radiomic features of habitat subregions are not just simple imaging quantitative indicators, but the phenotypic manifestation of the spatial heterogeneity of HER2-driven tumor microenvironment. In contrast, traditional radiomics models mostly extract global features from the entire tumor region, without fully accounting for the significant differences in cellular composition, microenvironment components, and molecular phenotypes among distinct functional regions within the tumor, but rather simplify highly heterogeneous tumor tissues into a single homogeneous entity (36). Such a global averaging feature representation fails to reveal the spatial heterogeneous structure driven by HER2 and cannot capture the local habitat information closely associated with molecular phenotypes. Therefore, it is significantly inferior to the precise radiomics analysis based on habitat subregions in terms of both biological interpretability and predictive performance.

However, in Task 2, which distinguishes between zero expression and low expression HER2 in young breast cancer patients, we found that the habitat model (AUC: 0.649, sensitivity: 43.6%, specificity: 74.7%) did not show better discriminatory performance compared to the whole-tumor model (AUC: 0.673, sensitivity: 47.3%, specificity: 73.4%). We hypothesize this is rooted in the minimal biological heterogeneity between HER2 zero and low expression breast cancers. Biologically, both HER2 zero and low expression tumors lack substantial HER2 amplification or overexpression, resulting in negligible differences in HER2-mediated tumor microenvironment remodeling (8). Without obvious spatial differences in pathobiological subregions between the two subtypes, the habitat model’s advantage of quantifying intratumoral heterogeneity is not manifested. In contrast, the whole-tumor radiomics model, which extracts global features, is not affected by such minimal subtype-specific heterogeneity and thus shows comparable or slightly better performance.

There are several limitations in this study. Firstly, since this is a retrospective study, the potential for selection bias cannot be ruled out. Secondly, although we included 375 cases of young breast cancer patients, there was an imbalance in sample distribution among the three groups of HER2 zero expression, low expression, and positive expression, which may affect the predictive performance of our model. Future studies should include a larger number of cases. Finally, although this model demonstrates certain clinical value in predicting HER2 expression status in young breast cancer patients, its generalization performance on external test sets remains to be improved due to factors such as MRI equipment and scanning parameters. Future research should focus on reducing the interference of device and parameter heterogeneity on the external generalization ability of the model, and further enhance its stability and applicability in multi-center and multi-device scenarios.

## Conclusions

In summary, our study compared the predictive performance of traditional radiomics models and habitat models for HER2 expression status in young breast cancer patients. The results confirmed that habitat imaging can effectively quantify intratumoral heterogeneity in young breast cancer patients and achieve accurate prediction of HER2 expression status. Our study not only advance the development of HER2 expression prediction in young breast cancer, but also provide reliable imaging evidence for clinical individualized anti-HER2 therapy and optimized treatment decision-making.

## Data Availability

The original contributions presented in the study are included in the article/**Supplementary Material**. Further inquiries can be directed to the corresponding author.

## Supplementary material

Supplementary Table 1 MRI acquisition parameters.

Supplementary Table 2 Selected feature chart.

Supplementary Figure 1 The performance of classification algorithms in task 1.

Supplementary Figure 2 The performance of classification algorithms in task 2.
