# Supplementary material for: Multiparametric MRI-based radiomics of whole-tumor and habitat regions for predicting HER2 status in young breast cancer: a two-center study
Source: Front Oncol. 2026 Mar 31;16:1760589. doi: 10.3389/fonc.2026.1760589 (PMC13076129; doi:10.3389/fonc.2026.1760589)
Supplement: Supplementary Table 1 — MRI acquisition parameters. [file Table1.docx]

**Table 1：**

**MRI acquisition parameters**

| **Sequence** | **Institution** | **TR**  **(ms)** | **TE**  **(ms)** | **Fat Suppression** | **FOV**  **(mm)** | **Slice Thickness**  **(mm)** | **Averages** | **​Matrix​** | **Parallel Imaging Factor** | **b-value**  **(s/mm²)** | **Acquisition Time​** |
| --- | --- | --- | --- | --- | --- | --- | --- | --- | --- | --- | --- |
| **DCE** | **I** | 4.50 | 1.60 | Q-fat sat | 340 | 1.0 | 1 | 448×448 | 2 | \ | 5:54 |
|  | **II** | 4.51 | 1.61 | Q-fat sat | 340 | 1.20 | 1 | 220×220 | 2 | \ | 6:56 |
| **DWI** | **I** | 8400 | 84 | SPAIR | 260 | 4.0 |  | 90×220 | 2 | 0 400 800 | 3:40 |
|  | **II** | 8400- | 85 | SPAIR | 320 | 4.0 | 3 | 300×448 | 2 | 50 800 | 2:56 |

Abbreviations: DCE=dynamic contrast enhancement; DWI=diffusion weighted imaging; TR=repetition time; TE=echo time; FOV=field of view
