# Supplementary material for: Multiparametric MRI-based radiomics of whole-tumor and habitat regions for predicting HER2 status in young breast cancer: a two-center study
Source: Front Oncol. 2026 Mar 31;16:1760589. doi: 10.3389/fonc.2026.1760589 (PMC13076129; doi:10.3389/fonc.2026.1760589)
Supplement: Supplementary Table 2 — Selected feature chart. [file Table2.docx]

# Feature chart

| **Model** | **Selected Feature** | **Coefficients** |
| --- | --- | --- |
| **Task 1: HER2-Negative vs HER2-Positive** | | |
| **Habitat Features** | wavelet-HHH_firstorder_Kurtosis_habitat_2_dce | -0.067280 |
|  | wavelet-LLL_glszm_GrayLevelNonUniformityNormalized_habitat_1_dce | -0.065107 |
|  | wavelet-HLH_glszm_GrayLevelNonUniformityNormalized_habitat_1_dwi | -0.064202 |
|  | wavelet-LLL_glszm_LargeAreaLowGrayLevelEmphasis_habitat_3_dce | 0.058105 |
|  | wavelet-LLL_firstorder_Kurtosis_habitat_1_dwi | 0.052643 |
|  | wavelet-LHL_glcm_Idn_habitat_1_dce | -0.038418 |
|  | wavelet-LHH_glszm_GrayLevelVariance_habitat_1_dce | 0.034257 |
|  | log-sigma-2-mm-3D_glszm_ZoneEntropy_habitat_3_dwi | 0.032622 |
|  | log-sigma-3-mm-3D_glcm_Correlation_habitat_3_dce | -0.024642 |
|  | wavelet-LHL_glcm_InverseVariance_habitat_1_dce | <0.000001 |
|  | wavelet-LLL_glszm_GrayLevelVariance_habitat_1_dce | <0.000001 |
|  | wavelet-LHL_glcm_DifferenceAverage_habitat_1_dce | <0.000001 |
|  | wavelet-LHL_glcm_Contrast_habitat_1_dce | <0.000001 |
| **Whole-tumor Features** | wavelet-HHH_firstorder_Kurtosis_dce | -0.085083 |
|  | log-sigma-3-mm-3D_gldm_DependenceNonUniformityNormalized_dwi | 0.062219 |
|  | wavelet-HLL_firstorder_Skewness_dwi | 0.060963 |
|  | wavelet-HHH_glszm_GrayLevelNonUniformityNormalized_dce | -0.056530 |
|  | wavelet-HLL_glcm_Imc1_dwi | -0.051145 |
|  | wavelet-LHL_glcm_Correlation_dce | -0.046203 |
|  | wavelet-HHL_firstorder_Skewness_dwi | -0.045330 |
|  | original_gldm_DependenceEntropy_dwi | -0.030699 |
|  | wavelet-LLL_glszm_SmallAreaLowGrayLevelEmphasis_dce | 0.030684 |
|  | original_shape_Maximum2DDiameterRow_dce | 0.030180 |
|  | wavelet-LHL_glcm_ClusterTendency_dce | -0.026645 |
|  | log-sigma-2-mm-3D_glrlm_ShortRunEmphasis_dce | 0.019611 |
|  | wavelet-HLL_glszm_GrayLevelNonUniformity_dce | 0.017300 |
|  | log-sigma-2-mm-3D_glrlm_ShortRunHighGrayLevelEmphasis_dce | 0.003409 |
|  | wavelet-HHH_glszm_GrayLevelVariance_dce | 0.000650 |
| **Clinical Features** | Ki-67 Index | <0.01 |
|  | Enhancement Pattern | 0.04 |
| **Task 2: HER2-Zero vs HER2-Low expression** | | |
| **Habitat Features** | wavelet-HHH_firstorder_Mean_habitat_1_dce | 0.107988 |
|  | wavelet-LLH_glcm_MCC_habitat_1_dwi | 0.097206 |
|  | wavelet-LHH_gldm_LowGrayLevelEmphasis_habitat_3_dwi | -0.073307 |
|  | wavelet-HLH_glszm_ZonePercentage_habitat_1_dwi | 0.065320 |
|  | original_glszm_SizeZoneNonUniformity_habitat_2_dce | 0.058299 |
|  | wavelet-LLL_firstorder_Skewness_habitat_1_dce | 0.048017 |
|  | original_shape_Elongation_habitat_3_dce | -0.045494 |
|  | wavelet-HLH_glcm_Imc2_habitat_1_dce | -0.035776 |
|  | wavelet-LHH_glszm_GrayLevelVariance_habitat_1_dwi | 0.028710 |
|  | wavelet-HLH_glcm_Imc1_habitat_1_dce | 0.022141 |
|  | original_firstorder_Skewness_habitat_1_dce | 0.015960 |
|  | wavelet-HHL_glszm_HighGrayLevelZoneEmphasis_habitat_3_dce | 0.014525 |
|  | log-sigma-2-mm-3D_firstorder_Skewness_habitat_1_dce | -0.005059 |
|  | wavelet-LHH_glszm_GrayLevelNonUniformityNormalized_habitat_1_dwi | -0.000149 |
|  | wavelet-LHH_gldm_HighGrayLevelEmphasis_habitat_3_dwi | <0.000001 |
| **Whole-tumor Features** | wavelet-HHL_glszm_SmallAreaHighGrayLevelEmphasis_dwi | 0.110998 |
|  | log-sigma-3-mm-3D_glcm_MCC_dwi | 0.084763 |
|  | wavelet-LHH_glszm_HighGrayLevelZoneEmphasis_dce | 0.076250 |
|  | original_glszm_SmallAreaLowGrayLevelEmphasis_dwi | -0.066389 |
|  | wavelet-LHH_glcm_Autocorrelation_dwi | 0.066025 |
|  | wavelet-LHL_glrlm_HighGrayLevelRunEmphasis_dwi | -0.055804 |
|  | wavelet-LHH_firstorder_Skewness_dwi | -0.034952 |
|  | log-sigma-3-mm-3D_firstorder_Skewness_dce | -0.031960 |
|  | wavelet-LHH_gldm_SmallDependenceLowGrayLevelEmphasis_dwi | 0.003191 |
|  | wavelet-LHL_glrlm_LowGrayLevelRunEmphasis_dwi | 0.002205 |
|  | wavelet-LHH_glszm_LowGrayLevelZoneEmphasis_dce | <0.000001 |
