# Supplementary figures and images for: Multiparametric MRI-based radiomics of whole-tumor and habitat regions for predicting HER2 status in young breast cancer: a two-center study
Source: Front Oncol. 2026 Mar 31;16:1760589. doi: 10.3389/fonc.2026.1760589 (PMC13076129; doi:10.3389/fonc.2026.1760589)

# External test set AUC for Task 1

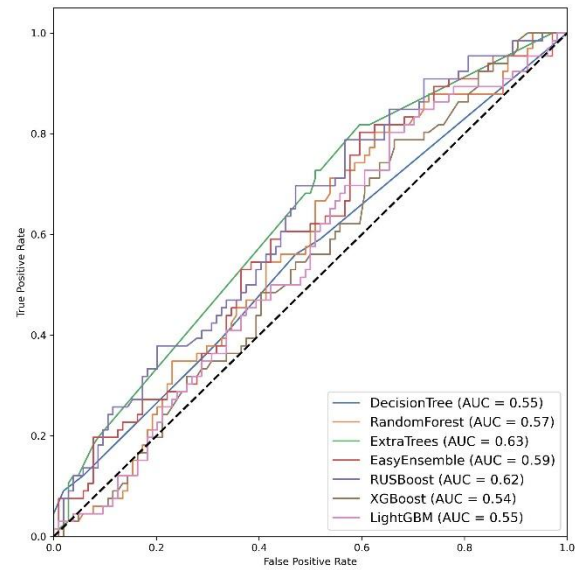

(a)

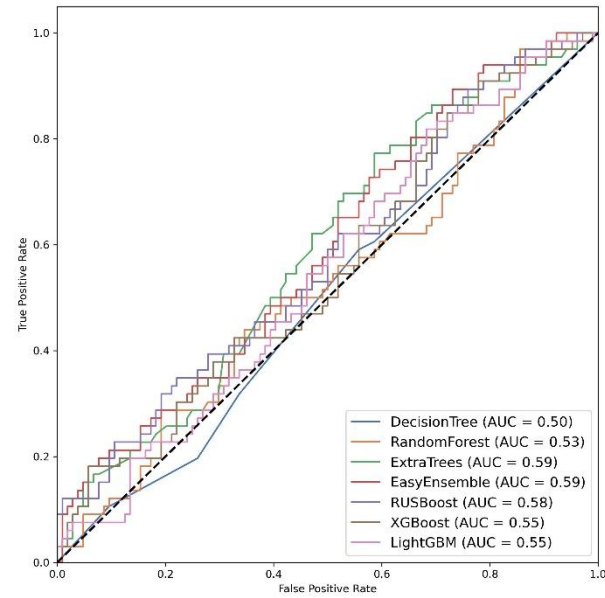

(b)

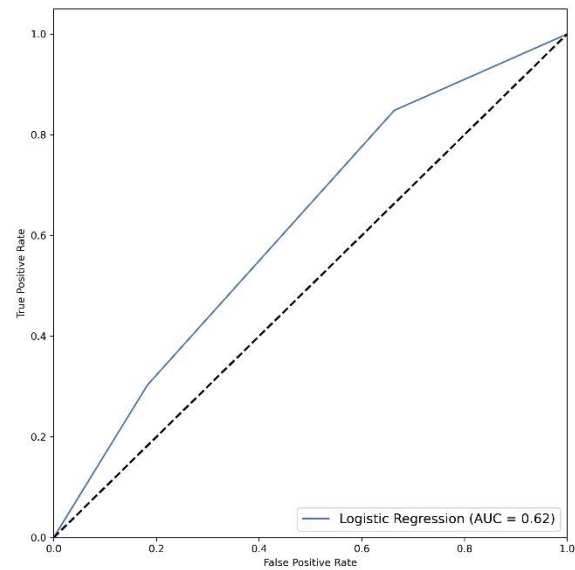

(c)

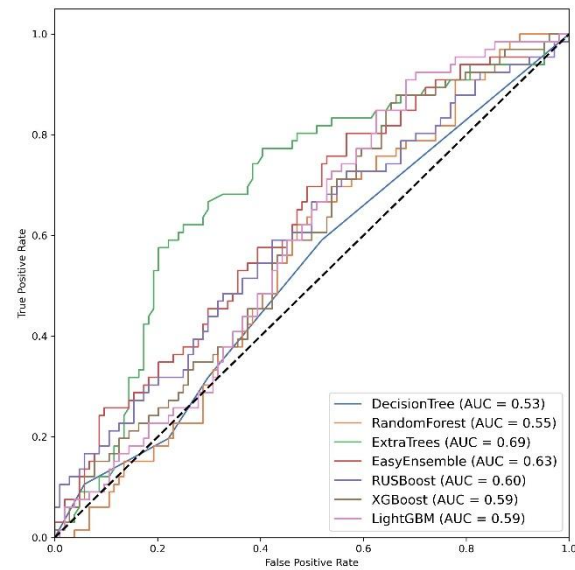

(d)

(a): Habitats  
(b): Whole Tumor  
(c): Clinical  
(d): Combined

Supplement: Supplementary Figure 1 — The performance of classification algorithms in task 1. [file DataSheet1.pdf]

## External test set AUC for Task 2

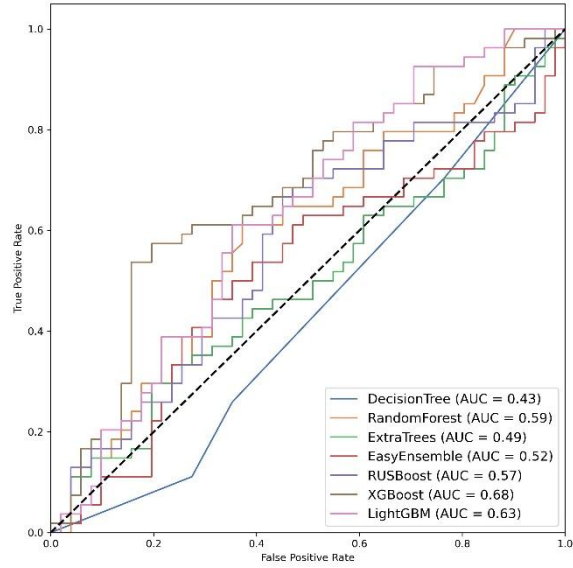

(a)

(a):Habitats

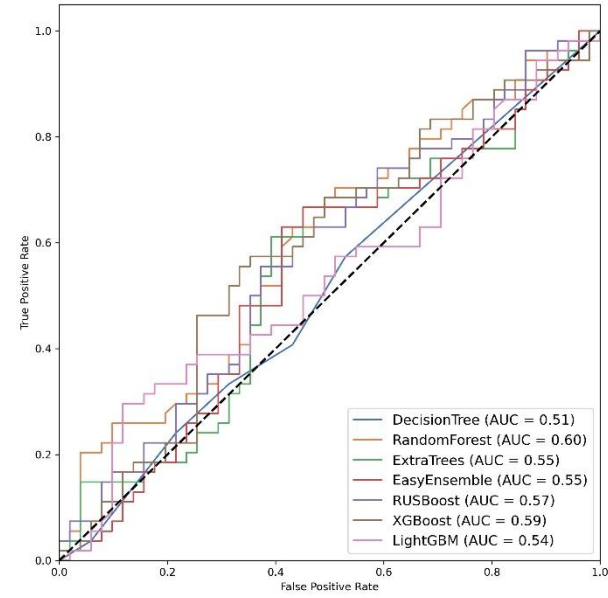

(b)

(b):Whole Tumor

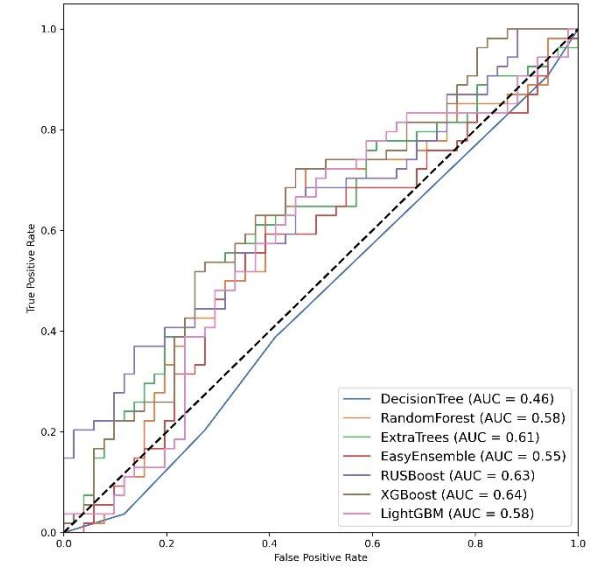

(c)

(c):Combined

Supplement: Supplementary Figure 2 — The performance of classification algorithms in task 2. [file DataSheet2.pdf]
